# Comparison of clinical efficacy and surgical safety among three bone graft modalities in spinal tuberculosis: a network meta-analysis

**DOI:** 10.1186/s13018-023-03848-9

**Published:** 2023-05-18

**Authors:** Jian Li, Xiuyu Qin, Jiani Wang, Wangzhe Yang, Junjun Bai, Jia Lv

**Affiliations:** 1 Department of Orthopaedics, Third Hospital of Shanxi Medical University, Shanxi Bethune Hospital, Shanxi Academy of Medical Sciences, Tongji Shanxi Hospital, Taiyuan, 030032 China; 2 Department of Orthopaedics, The Second Hospital of Shanxi Medical University, Taiyuan, 030001 China; 3 Department of Paediatric Medicine, Shanxi Medical University, Taiyuan, 030001 China

**Keywords:** Autogenous granular bone graft, Autogenous massive bone graft, Titanium mesh bone graft, Spinal tuberculosis, Network meta-analysis

## Abstract

**Background:**

Autogenous granular bone graft (AG), autogenous massive bone graft (AM), and titanium mesh bone graft (TM) are the three commonly utilized bone implant methods for spinal tuberculosis. However, the gold standard is still controversial. Therefore, this study aimed to compare the clinical efficacy and surgical safety of three primary bone graft modalities.

**Methods:**

For systematic literature review, several databases, including PubMed, Embase, and Web of Science, were searched up to December 2022. Stata (version 14.0) was employed for data analysis.

**Results:**

Our network meta-analysis included 517 patients from 7 articles whose qualities are acceptable based on our quality assessment criteria. In direct comparison, AG was associated with a shorter operation time (MD = 73.51; CI 30.65–116.37) and a lesser blood loss (MD = 214.30; CI 7.17–421.44) than AM. TM had fewer loss of Cobb angle than AG (MD = 1.45; CI 0.13–2.76) and AM (MD = 1.21; CI 0.42–1.99). Compared with AG, TM (MD = 0.96; CI 0.06–1.87) was related to a shorter bone graft fusion time. In indirect comparison, for the clinical parameters, the rank of CRP (from best to worst) was as follows: TM (58%) > AM (27%) > AG (15%), the rank of ESR (from best to worst) was as follows: AG (61%) > AM (21%) > TM (18%), and the rank of VAS (from best to worst) was as follows: AG (65%) > TM (33%) > AM (2%). In the aspect of surgical data, what is noteworthy is that AG showed less blood loss [AG (93%) > TM (6%) > AM (1%)], operative time [AG (97%) > TM (3%) > AM (0)], and complications [AG (75%) > TM (21%) > AM (4%)] than AM and TM. As for imaging parameters, the rank of the loss of Cobb angle (from best to worst) was as follows: TM (99%) > AM (1%) > AG (0). Moreover, TM showed a shorter bone graft fusion time than AM and AG: TM (96%) > AM (3%) > AG (1%).

**Conclusions:**

The results indicated that AG might be the optional treatment for spinal tuberculosis owing to the outcomes of surgical safety. Moreover, TM is another right choice which can significantly reduce the loss of Cobb angle and shorten bone graft fusion time with long‐term follow‐up.

## Background

Tuberculosis is a common chronic respiratory infectious disease caused by *Mycobacterium tuberculosis* and is one of the main threats to human health. Tuberculosis usually infects the lungs. However, tuberculosis can travel through the blood from the lungs into other organs of the whole human body, resulting in extrapulmonary tuberculosis. The most common form of extrapulmonary tuberculosis is bone and joint tuberculosis, which occurs mainly in the spine [1]. Spinal tuberculosis is the most frequent type of bone and joint tuberculosis, occurring in about 50% of cases [2, 3], especially involve the lower thoracic and upper lumbar spine. Late or neglected spinal tuberculosis can result in severe spinal kyphosis due to bony destruction and vertebral collapse. Kyphosis can lead to back pain, increased instability, vertebral translations, and neurological injury, which seriously affects the life of patients [4].

Most patients with spinal tuberculosis will recover with pharmaceutical treatment. However, surgical treatment is needed to treat patients with severe tuberculosis toxicity and compression symptoms. The main purpose of the operation is to relieve the symptoms of the spinal cord and nerve root compression and reshape the stability of the spine [5, 6]. The surgical procedures often include focus debridement, bone graft fusion, internal fixation, and so on. Among them, bone grafting plays an important role in the reconstruction of vertebral height and spinal stability.

At present, the commonly used bone grafting materials include autogenous granular bone graft (AG), autogenous massive bone graft (AM), titanium mesh bone graft (TM) [7]. But the standard type of bone grafting material for spinal tuberculosis is still controversial. A growing number of studies have investigated the efficacy of different methods of bone grafting. For example, Xu et al. [8] had demonstrated that AG is more effective in reducing postoperative complications and shortening surgical trauma compared to AM. The results of application for TM and AM in spinal tuberculosis show that titanium mesh outperformed autogenous massive bone in operation duration, blood loss, VAS, loss of angular correction, and surgical complications [9]. However, we reviewed the pieces of literature and found that evidence-based medical evidence for bone grafting in spinal tuberculosis is difficult to identify. So far there is only a meta-analysis of AM and TM in the treatment of spinal tuberculosis, which does not include AG unfortunately [10]. To investigate the clinical efficacy and surgical safety of different bone grafting modalities (AG, AM, and TM) for the treatment of spinal tuberculosis, a network meta-analysis was performed to assess the effectiveness and guide the choice of bone grafts in the future.

## Methods

### Search strategies and selection criteria

The network meta-analysis was designed and performed in accordance with the Preferred Reporting Items for Systematic Reviews and Meta-Analyses (PRISMA) guidelines [11]. We conducted literature retrieval from the earliest record to December 2022, using the following electronic databases: PubMed, Embase, and Web of Science. The keywords for the articles search were “spinal tuberculosis,” “thoracolumbar tuberculosis,” “lumbar tuberculosis,” “thoracic tuberculosis,” “Titanium mesh,” “Autogenous bone,” “Allogenic bone,” “Allograft bone,” and “artificial bone.” The language for searching publications is limited to English. Two authors (Jian Li and Xiuyu Qin) checkout the articles independently and resolved the conflict options by discussing with the corresponding author (Jia Lv).

Studies that met the following criteria were selected for network meta-analysis: (1) randomized controlled trials (RCT) or retrospective cohort studies (RS); (2) patients were diagnosed with spinal tuberculosis (including the thoracic and lumbar, but not the cervical) and had surgical indications for decompression and bone grafting; (3) the clinical efficacy and surgical safety among AG (nonstructural autograft), AM (structural autograft), and/or TM (titanium mesh allograft) treatment methods was compared; (4) including at least one of the following indicators: surgical data (operation time, blood loss, or hospital stay), clinical parameters [visual analog scale (VAS)], erythrocyte sedimentation rate (ESR), or C-reactive protein (CRP), or imaging parameters (loss of Cobb angle or bone graft fusion time). The exclusion criteria were as follows: (1) Different studies contain the same patients; in this case, only the study showed the most outcome indicators were included; (2) articles only contain animal experiments, case reports, reviews, conference reports, or comments; (3) studies with only unavailable data.

### Data extraction and quality assessment

Two authors (Jian Li and Xiuyu Qin) extracted the available information from the original articles. The information included (1) study characteristics: first author, publication date, study site, study design, follow-up time, and sample size, etc.; (2) intervention measures: AG, AM, or/and TM; (3) clinical parameters: ESR, CRP, and VAS; (4) surgical data: operation time, blood loss, and complications (cerebrospinal fluid leakage, drug-induced liver dysfunction or kidney dysfunction, postoperative infection, sinus formation, bacterial or tuberculous meningitis, intervertebral infection, donor-site infection and long-term chronic pain, failure of pedicle nail, or fixation rod); (5) imaging parameters: loss of Cobb angle and bone graft fusion time. For an article with insufficient data, we will try our best to contact the authors of the original literature by email to request supplementary information. If there is no response, the study will be canceled.

The quality of RS was assessed using the Newcastle–Ottawa scale (NOS) [12], which had 8 items divided into 3 categories with a total of 9 points: selection (4 points), comparability (2 points), and expose (3 points). Generally speaking, articles with scores of 6 and above are considered high-quality research. Two authors (Jian Li and Xiuyu Qin) independently evaluated and summarized the quality of each included study. The discrepancies between authors were reconciled through discussion with the corresponding author (Jia Lv).

### Statistical analysis

Stata (version 14.0) was employed for network meta-analysis. Firstly, the local inconsistency test of included data is checked using the node-splitting method. In the above inconsistency test, the inconsistency model was considered insignificant at *P* > 0.05, and the consistency modeling analysis tool was adopted for the next step of data analysis. Secondly, the Bayesian random effects model was utilized to merge the assessments of direct and indirect treatment comparisons, and the interventions were ranked using the tool of consistency model. Finally, the differential transplants were sorted using the surface under the cumulative sorting curve (SUCRA). The larger the SUCRA is, the better rank the bone graft will be. In the above analysis, odds ratio (OR) with 95% confidence interval (CI) will be applied for dichotomous variables, while mean difference (MD) with 95% CI will be estimated for continuous outcomes.

## Results

### Characteristics of included studies

**Fig. 1:**
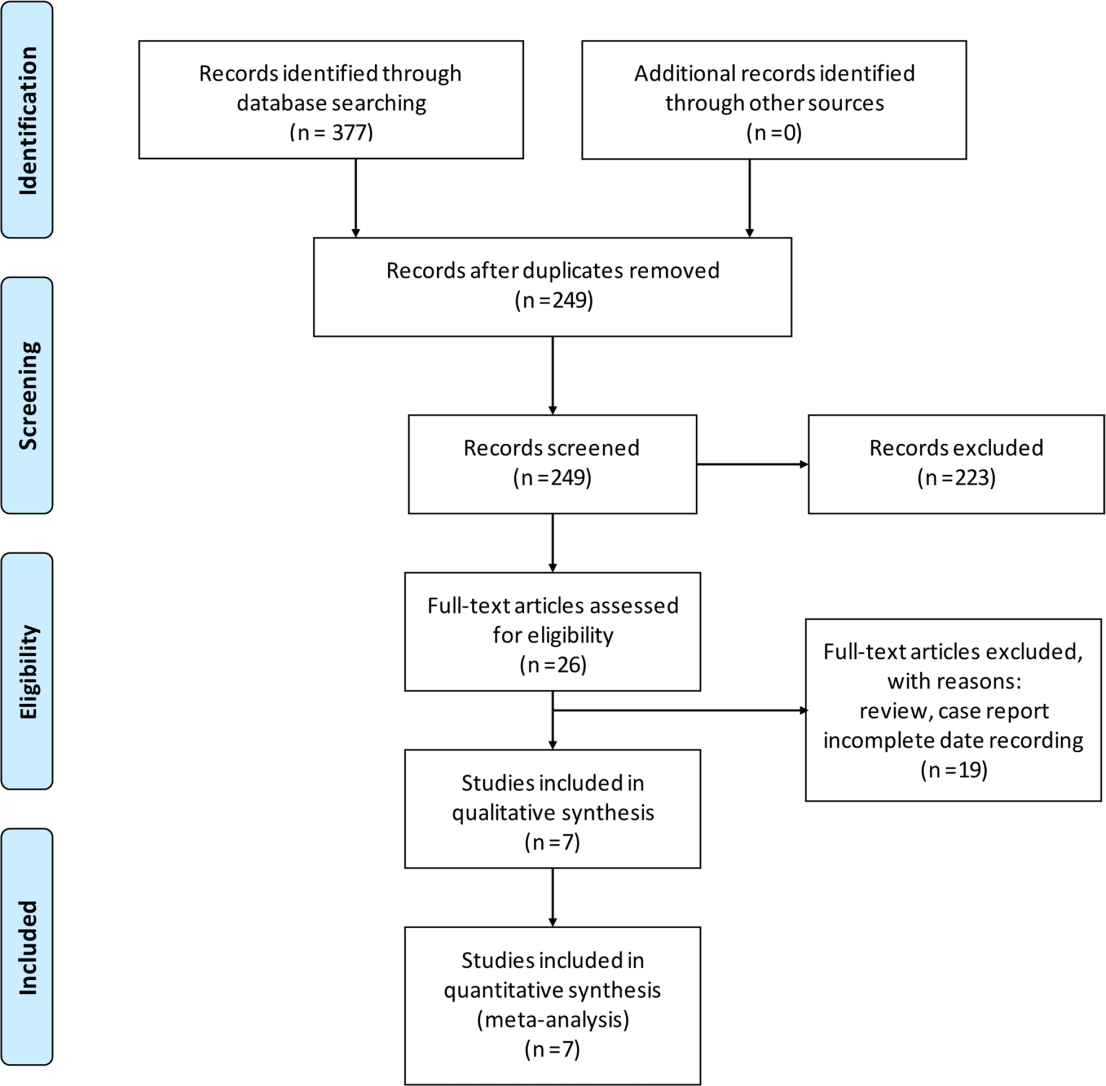
PRISMA flow diagram

As shown in Fig. 1, a total of 377 articles were initially retrieved using the established search strategy. A total of 128 duplicated literature were excluded. Another 223 articles were excluded after evaluating the titles and abstracts. After screening the full text, 7 literatures involving 517 patients (AG: 65 cases, AM: 247 cases, TM: 205 cases) were analyzed in the current network meta-analysis [5, 7–9, 13–15]. The basic characteristics of these studies are summarized in Table 1.

**Table 1:** Characteristics of the included studies in network meta-analysis

Study ID	Study design	Level of TB	Group	Sex (M/F)	Age (years)	Sample sizes	Follow-up (months)	Results
He et al. [7]	RS	Thoracic	AM	12/16	46.32 ± 14.07	28	24	①②③④⑤⑥⑧
			TM	11/15	49.97 ± 16.36	36		
Gao et al. [5]	RS	Thoracic and lumbar	AM	9/16	41.4 ± 14.3	25	35.5	①②③④⑤⑥⑦⑧
			TM	13/12	39.2 ± 14.2	25		
Yin et al. [15]	RS	Lumbar	AM	11/8	55.5 ± 12.6	19	47.3 ± 8.1	④⑤⑥⑦⑧
			TM	10/7	49.9 ± 15.4	17		
Wu et al. [9]	RS	Thoracic and lumbar	AM	33/27	48.0 ± 16.5	60	60	①③⑤⑦
			TM	55/31	53.1 ± 18.8	86		
Liu et al. [13]	RS	Lumbar	AG	11/11	43.9 ± 12.1	22	21.6 ± 5.7	①②④⑤⑥⑦⑧
			AM	19/17	40.5 ± 16.5	36	22.3 ± 6.2	
Xu et al. [8]	RS	Lumbar	AG	23/20	47.4 ± 12.4	43	28.7 ± 4.3	①②③④⑤⑥⑦⑧
			AM	22/17	48.2 ± 12.0	39		
Xu et al. [14]	RS	Lumbar	AM	24/17	47.6 ± 13.3	41	76.5 ± 11.2	③④⑤⑥⑦⑧
			TM	24/16	49.4 ± 12.3	40		

*TB* tuberculosis, *RS* retrospective cohort study, *QAS* quality assessment score, *AM* autogenous massive bone graft, *AG* autogenous granular bone graft, *TM* titanium mesh bone graft, ① CRP, ② ESR, ③ VAS, ④ blood loss, ⑤ operation time, ⑥ complications, ⑦ loss of Cobb angle, ⑧ bone graft fusion time

The score of quality assessment for the 7 included studies is shown in Table 2. No high-risk studies were identified in the included studies.

**Table 2:** The Newcastle–Ottawa scale (NOS) was used for assessing the quality of included studies

Items	He et al. [7]	Gao et al. [5]	Yin et al. [15]	Wu et al. [9]	Liu et al. [13]	Xu et al. [8]	Xu et al. [14]
Is the case definition adequate	★	★	★	★	★	★	★
Representativeness of the cases	★	★	★	★	★	★	★
Selection of controls	★	★	–	★	★	–	★
Definition of controls	★	★	★	★	★	★	★
Comparability							
Study controls for the most important factor	★	★	★	★	★	★	★
Study controls for any additional factor	★	–	★	★	–	★	–
*Exposure*							
Ascertainment of exposure	★	★	★	★	★	★	★
Same method of ascertainment for cases and controls	★	★	★	★	★	★	★
Nonresponse rate	–	–	–	–	–	–	–
Total scores	8	7	7	8	7	7	7

### Clinical parameters

**Fig. 2:**
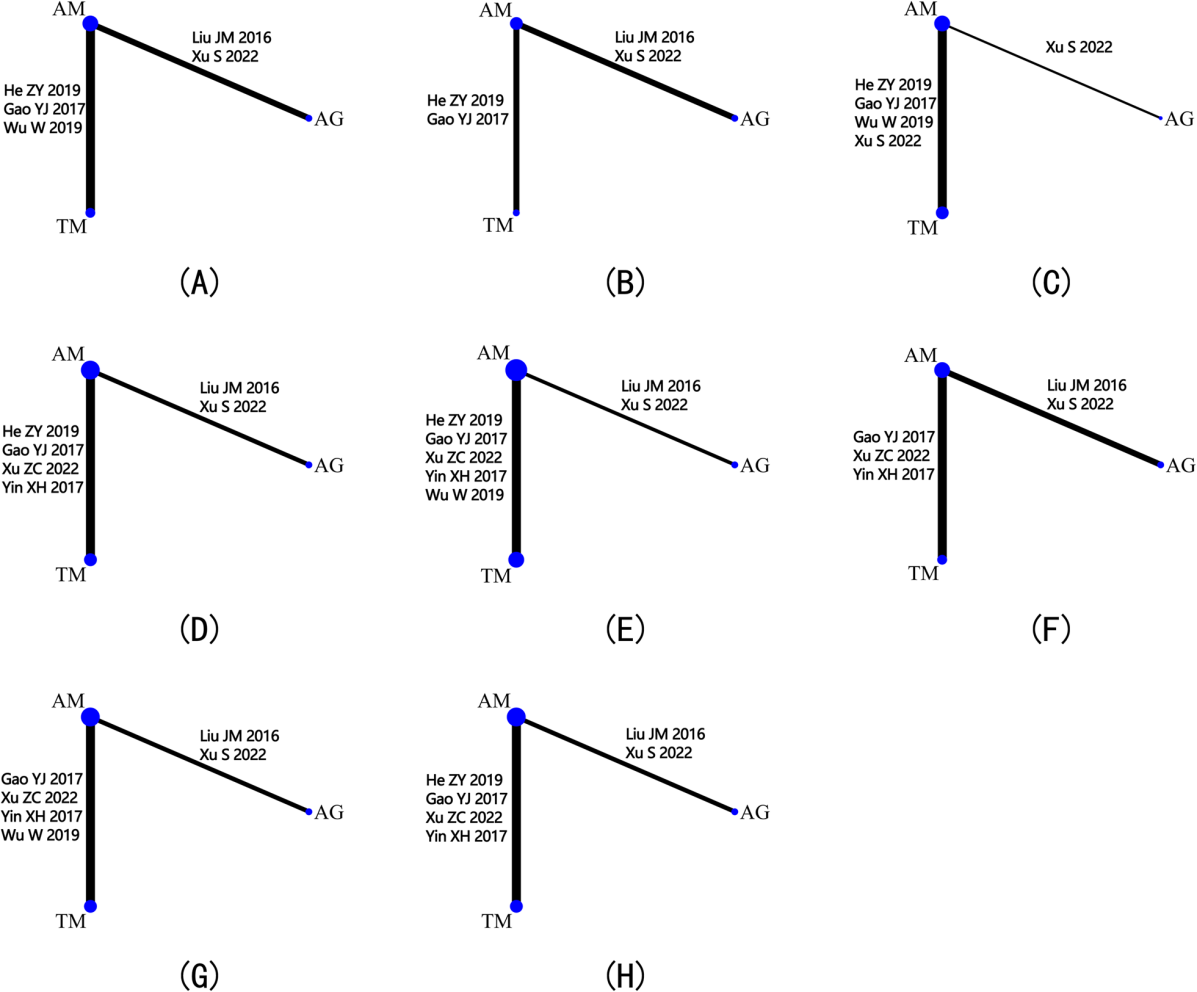
Network of different treatments. **A** Comparison for CRP; **B** comparison for ESR; **C **comparison for VAS; **D** comparison for blood loss; **E** comparison for operation time. **F** Comparison for complications; **G** comparison for loss of Cobb angle; **H** comparison for bone graft fusion time

**Fig. 3:**
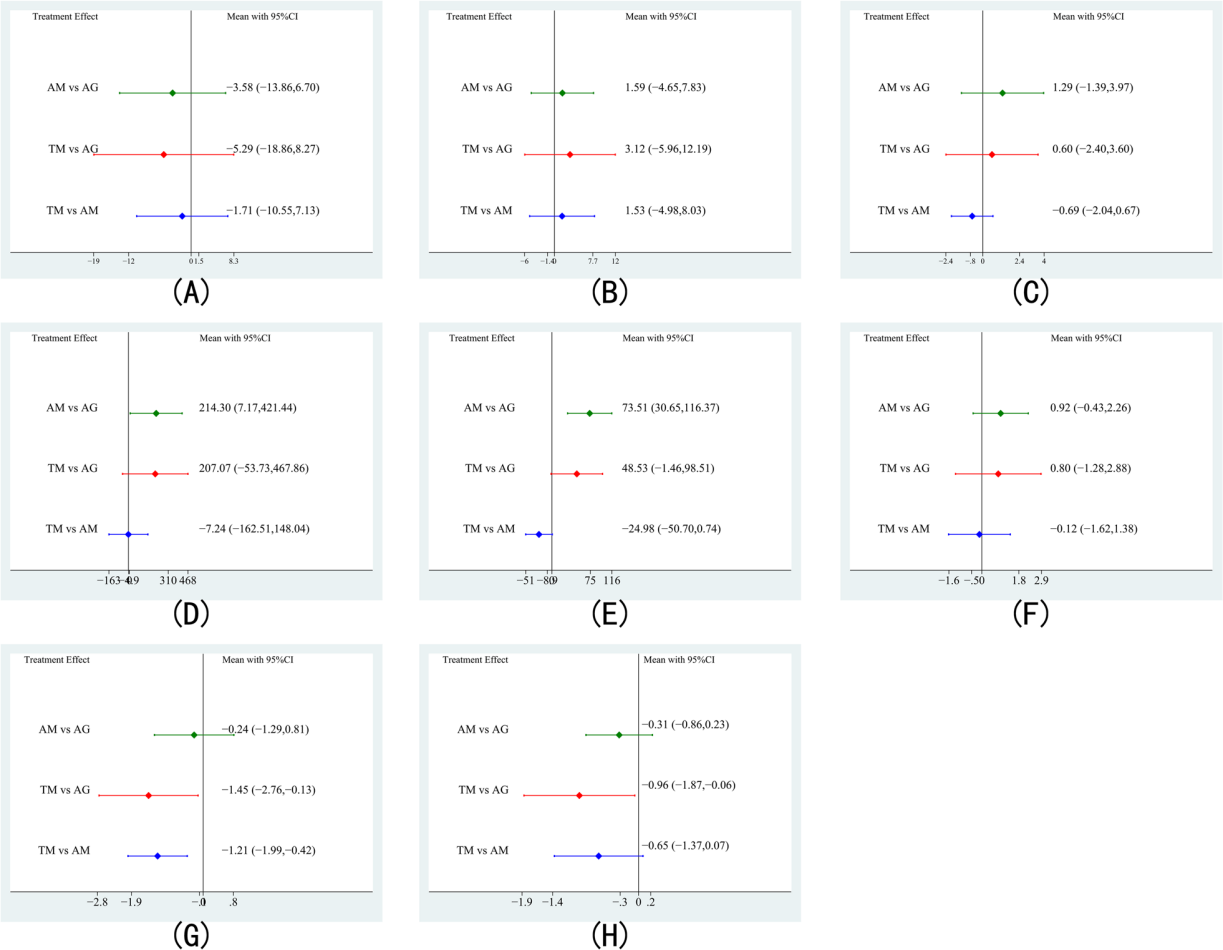
Forest plot of direct comparison between different treatments. **A** Comparison for CRP; **B** comparison for ESR; **C** comparison for VAS; **D** comparison for blood loss; **E** comparison for operation time. **F** Comparison for complications; **G** comparison for loss of Cobb angle; **H** comparison for bone graft fusion time

**Fig. 4:**
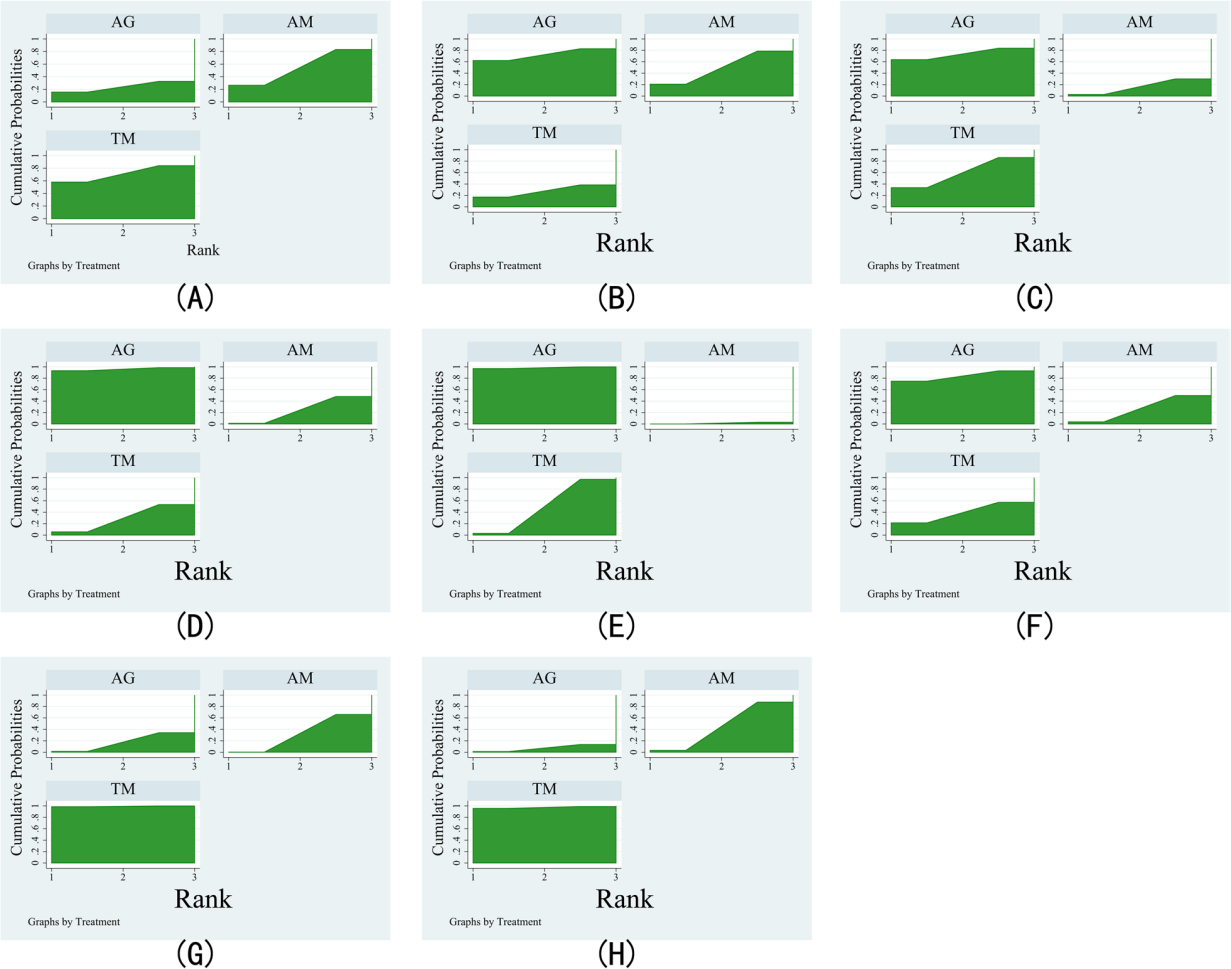
Plot of the surface under the cumulative ranking curves for different treatments. **A** Comparison for CRP; **B** comparison for ESR; **C** comparison for VAS; **D** comparison for blood loss; **E** comparison for operation time. **F** Comparison for complications; **G** comparison for loss of Cobb angle; **H** comparison for bone graft fusion time

Data about the clinical parameters, including postoperative CRP, ESR, and VAS, were extracted and analyzed by network meta-analysis. For CRP, 3 studies consisting of 260 patients compared the outcomes of AM with those of TM. Moreover, the comparison of the outcomes of AM and AG was conducted on 140 patients involved in 2 studies (Fig. 2A). No significant difference was detected in CRP among the three treatments (Fig. 3A). Based on the ranking of treatments, TM had the highest probability of being the best (58%) treatment, followed by AG (27%) and AM (15%) (Fig. 4A and Table 3).

**Table 3:** Percentage plot of the rank probabilities among different treatments

Items	AG	AM	TM
CRP	0.146	0.268	0.586
ESR	0.615	0.210	0.175
VAS	0.646	0.025	0.329
Blood loss	0.933	0.011	0.057
Operation time	0.970	0	0.030
Complications	0.749	0.037	0.213
Loss of Cobb angle	0.002	0.013	0.985
Bone graft fusion time	0.011	0.032	0.957

Number in the table indicates the odds of being ranked first

For ESR, 3 studies consisted of 196 patients and reported AM versus TM, 2 studies consisted of 140 patients and reported AM versus AG, and 2 studies consisted of 114 patients and reported AM versus TM (Fig. 2B). There is no significant distinction between the three treatment groups (Fig. 3B). The treatment ranking results showed that AG was most likely to become the best (61%) treatment method, followed by AM (21%) and TM (18%) (Fig. 4B and Table 3).

For VAS, 4 studies consisted of 341 patients and reported AM versus TM, and one study consisted of 82 patients and reported AM versus AG (Fig. 2C). There was no significant difference among the three treatment groups (Fig. 3C). Ranking the treatments by analyzing their probability of being the best treatment demonstrated that AG has the most potential to be the best (65%) treatment, followed by TM (33%) and AM (2%) (Fig. 4C and Table 3).

### Surgical data

Surgical data, including blood loss, operation time, and complications, were also employed for evaluating the performance of these three methods. For blood loss, 4 studies consisted of 231 patients and reported AM versus TM, and 2 studies consisted of 140 patients and reported AM versus AG (Fig. 2D). In direct comparison, AG performed better than AM in reducing the blood loss (Fig. 3D). According to the ranking of treatments, AG had the highest probability of being the best (93%), followed by TM (6%) and AM (1%) (Fig. 4D and Table 3).

For operation time, 5 studies consisted of 377 patients and reported AM versus TM, and 2 studies consisted of 140 patients and reported AM versus AG (Fig. 2E). Compared with AM, AG exhibited a shorter operation time (Fig. 3E). As indicated by the treatment ranking, AG has the highest probability (97%), followed by TM (3%) and AM (0) (Fig. 4E and Table 3).

For complications, 3 studies consisted of 167 patients and reported AM versus TM, and 2 studies consisted of 140 patients and reported AM versus AG (Fig. 2F). None of the treatment groups showed a significant difference (Fig. 3F). The results of the treatment ranking showed that AG had the highest probability of being the best (75%), followed by TM (21%) and AM (4%) (Fig. 4F and Table 3).

### Imaging parameters

Imaging parameters included loss of Cobb angle and bone graft fusion time. For loss of Cobb angle, 4 studies consisted of 313 patients and reported AM versus TM, and 2 studies consisted of 140 patients and reported AM versus AG (Fig. 2G). Meanwhile, AG and AM have more loss of Cobb angle than TM (Fig. 3G). As a result of the treatment ranking, TM showed the highest probability of being the most effective (99%) method, followed by AM (1%) and AG (0) (Fig. 4G and Table 3).

For bone graft fusion time, 4 studies consisted of 231 patients and reported AM versus TM, and 2 studies consisted of 140 patients and reported AM versus AG (Fig. 2H). In comparison with AG, TM performed better in bone graft fusion time (Fig. 3H). Treatment ranking results showed that TM was most likely to be the best (96%), followed by AM (3%) and AG (1%) (Fig. 4H and Table 3).

### Consistency tests

To evaluate the reliability of this network meta-analysis, the local consistency test of included data was performed. As shown in Table 4, a local consistency test (node-splitting analysis) revealed no significant difference between the direct and indirect evidence in any of the involved outcomes (all *P* > 0.05). The above results suggested that this network meta-analysis can be performed reliably.

**Table 4:** Local consistency model test between different treatments

Items	AG versus AM/AM versus TM
CRP	0.978
ESR	0.992
VAS	0.950
Blood loss	0.952
Operation time	0.908
Complications	0.988
Loss of Cobb angle	0.917
Bone graft fusion time	0.989

Number is *P* value in the table

## Discussion

Bone grafting is a critical step in surgical treatments of spinal tuberculosis, which helps to re-establish the stability of the spine after debridement [16]. Bone implant modalities in spinal tuberculosis include AG, AM, and TM. Previous studies have investigated the difference in surgical outcomes among different bone grafting procedures by Du, Srivastava, et al. [17–20], whereas the meta-analysis in this field remains limited. To the best of our knowledge, among the above research, only one study reported by He et al. [10] compared the surgical effects of AM and TM using a meta-analysis. Their findings suggest that both TM and AM alone are effective for the treatment of thoracolumbar spinal tuberculosis based on safety, but nonsupport the superiority of TM or AM reported in previous studies. However, they did not include the AG, which led to incomplete results. Here, we directly compared the clinical efficacy and surgical safety among AG, AM, and TM using network meta-analysis and revealed that AG has much better outcomes than AM and TM in operation time, blood loss, and complications. Moreover, TM has its superiority in reducing the loss of Cobb angle and shortening bone graft fusion time with long‐term follow‐up. The results of this study provided a comprehensive reference for clinicians to select suitable bone implants for spinal tuberculosis.

Different bone grafting methods have distinct effects on surgical outcomes. Among the three bone grafting approaches analyzed in this study, as predicted, there were no significant differences in postoperative ESR and CRP. As a result of pain in the area of bone extraction, patients with AM had a higher VAS score than those with AG and TM [21]. It is known that previously the most common form of osseous implant is the AM, which can provide good strength and fusion due to its superior osteoconduction and osteoinduction [22]. However, the surgical complications increase in this method because it needs more additional surgical procedures, the most common among which is longer operative time, increased intraoperative bleeding, pain, and fracture at the iliac bone graft harvest site. Increasing evidence has demonstrated that AG has an advantage in reducing complications, operative time, and intraoperative blood loss [17], which is also consistent with the results in our present meta-analysis.

When it comes to spinal fusion, surprisingly, we found that TM showed a fewer fusion time of bone graft than AM and AG in the treatment of spinal tuberculosis. It is widely acknowledged that a stable fusion created by bone grafting is essential for the long-term efficacy of surgical treatment. Some researchers have revealed that titanium mesh implants filled with cancellous bone have been observed to have a greater contact area with the endplate, which can significantly enhance anterior column stability and thus improve fusion rates [23]. Meanwhile, we discovered that TM has a unique advantage in reducing postoperative Cobb angle loss when compared with AG and AM, probably attributing to a reduction of bone resorption [9]. This result is akin to previous clinical studies demonstrating that intervertebral titanium mesh bone graft performed better in maintaining lordosis and preventing collapse than autogenous bone graft [14]. Based on these results, TM is likely to have a promising future in correcting the deformity and maintaining spinal stability.

Recently, several bone graft substitutes have been developed, including recombinant human Bone Morphogenetic Proteins (rhBMPs), transverse process bone graft, and ceramics, which have been increasingly used in operations [24]. Some studies insist that bone graft substitutes are feasible under specific conditions, with some security risks [25], while others suggest that the new method of bone graft, such as transverse process bone graft, is a safe grafting way. Further research and refinement are needed in this field.

Several limitations of the present study should be considered. First, most samples included in this publication are from China, which may induce some bias. Second, the nature of the included studies, such as a retrospective and single-center study with a relatively small sample size, may lead to statistical bias.

## Conclusions

The AG has better outcomes than AM and TM in operation time, blood loss, and complications in treatment of spinal tuberculosis. Moreover, TM has a unique advantage in reducing postoperative Cobb angle loss and shortening bone graft fusion time with long‐term follow‐up. All in all, the most appropriate bone grafting method should be evaluated on a case-by-case basis in clinical practice.

## Data Availability

All data generated or analyzed during this study are included in this published article.

## Abbreviations

AG: Autogenous granular bone graft
AM: Autogenous massive bone graft
TM: Titanium mesh bone graft
MD: Mean difference
CI: Confidence interval
OR: Odds ratio
CRP: C-reactive protein
ESR: Erythrocyte sedimentation rate
VAS: Visual analog scale
PRISMA: Preferred Reporting Items for Systematic Reviews and Meta-Analysis
RCT: Randomized controlled trials
RS: Retrospective cohort studies
NOS: Newcastle–Ottawa scale
SUCRA: Surface under the cumulative sorting curve
rhBMPs: Recombinant human Bone Morphogenetic Proteins

## References

[1] Fan J, An J, Shu W (2020). Epidemiology of skeletal tuberculosis in Beijing, China: a 10-year retrospective analysis of data. Eur J Clin Microbiol Infect Dis.

[2] Rajasekaran S, Khandelwal G (2013). Drug therapy in spinal tuberculosis. Eur Spine J.

[3] Gautam MP, Karki P, Rijal S (2005). Pott's spine and paraplegia. JNMA J Nepal Med Assoc.

[4] Liu C, Lin L, Wang W (2016). Long-term outcomes of vertebral column resection for kyphosis in patients with cured spinal tuberculosis: average 8-year follow-up. J Neurosurg Spine.

[5] Gao Y, Ou Y, Deng Q (2017). Comparison between titanium mesh and autogenous iliac bone graft to restore vertebral height through posterior approach for the treatment of thoracic and lumbar spinal tuberculosis. PLoS ONE.

[6] Wu P, Wang XY, Li XG (2015). One-stage posterior procedure in treating active thoracic spinal tuberculosis: a retrospective study. Eur J Trauma Emerg Surg.

[7] He Z, Tang K, Gui F (2019). Comparative analysis of the efficacy of a transverse process bone graft with other bone grafts in the treatment of single-segment thoracic spinal tuberculosis. J Orthop Surg Res.

[8] Xu S, Zhang S, Wang G (2022). Comparison of clinical and radiological outcomes of local morselized bone grafts and structural iliac bone grafts in the treatment of lumbar tuberculosis with posterior-only surgery. BMC Surg.

[9] Wu W, Wang S, Li Z (2021). Posterior-only approach with titanium mesh cages versus autogenous iliac bone graft for thoracic and lumbar spinal tuberculosis. J Spinal Cord Med.

[10] He Z, Ou Y, Hou B (2020). A meta-analysis of the safety and effectiveness of titanium mesh versus bone graft alone for the treatment of thoracolumbar tuberculosis. Eur Spine J.

[11] Page MJ, McKenzie JE, Bossuyt PM (2021). The PRISMA 2020 statement: an updated guideline for reporting systematic reviews. BMJ.

[12] Jadad AR, Moore RA, Carroll D (1996). Assessing the quality of reports of randomized clinical trials: is blinding necessary?. Control Clin Trials.

[13] Liu JM, Chen XY, Zhou Y (2016). Is nonstructural bone graft useful in surgical treatment of lumbar spinal tuberculosis?: A retrospective case-control study. Medicine (Baltimore).

[14] Xu Z, Wang X, Zhang Z (2022). A comparison of three bone graft struts for interbody fusion using a posterior approach for lower lumbar spinal tuberculosis in adults: a midterm follow-up study. BMC Musculoskelet Disord.

[15] Yin XH, Liu ZK, He BR (2017). Single posterior surgical management for lumbosacral tuberculosis: titanium mesh versus iliac bone graft: a retrospective case-control study. Medicine (Baltimore).

[16] Xu Z, Wang X, Liu Z (2020). One-stage posterior debridement and single-segment interbody fusion for treating mono-segmental lumbar and lumbosacral spinal tuberculosis in adults following minimum 5-year follow-up. J Orthop Surg Res.

[17] Du X, Ou YS, Xu S (2020). Comparison of three different bone graft methods for single segment lumbar tuberculosis: a retrospective single-center cohort study. Int J Surg.

[18] Wang B, Hua W, Ke W (2020). The efficacy of allograft bone using titanium mesh in the posterior-only surgical treatment of thoracic and thoracolumbar spinal tuberculosis. BMC Surg.

[19] Srivastava SK, Marathe NA, Bhosale SK (2020). Outcome analysis of anterior reconstruction with rib grafts in tuberculosis of the thoracic spine. Asian J Neurosurg.

[20] Zhang HQ, Li M, Wang YX (2019). Minimum 5-year follow-up outcomes for comparison between titanium mesh cage and allogeneic bone graft to reconstruct anterior column through posterior approach for the surgical treatment of thoracolumbar spinal tuberculosis with kyphosis. World Neurosurg.

[21] Tang K, Li J, Huang T (2020). Clinical efficacy of three types of autogenous bone grafts in treatment of single-segment thoracic tuberculosis: a retrospective cohort study. Int J Med Sci.

[22] Sundararaj GD, Amritanand R, Venkatesh K (2011). The use of titanium mesh cages in the reconstruction of anterior column defects in active spinal infections: can we rest the crest?. Asian Spine J.

[23] Li Z, Wu W, Chen R (2019). Could allograft bones combined with poly-ether-ether-ketone cages or titanium mesh cages be an alternative grafting method in the management of cervical spinal tuberculosis?. World Neurosurg.

[24] Hsu WK, Nickoli MS, Wang JC (2012). Improving the clinical evidence of bone graft substitute technology in lumbar spine surgery. Glob Spine J.

[25] Grabowski G, Cornett CA (2013). Bone graft and bone graft substitutes in spine surgery: current concepts and controversies. J Am Acad Orthop Surg.

