# Correction: Comparison of clinical efficacy and surgical safety among three bone graft modalities in spinal tuberculosis: a network meta-analysis

**DOI:** 10.1186/s13018-023-03876-5

**Published:** 2023-05-31

**Authors:** Jian Li, Xiuyu Qin, Jiani Wang, Wangzhe Yang, Junjun Bai, Jia Lv

**Affiliations:** 1 Department of Orthopaedics, Third Hospital of Shanxi Medical University, Shanxi Bethune Hospital, Shanxi Academy of Medical Sciences, Tongji Shanxi Hospital, Taiyuan, 030032 China; 2 Department of Orthopaedics, The Second Hospital of Shanxi Medical University, Taiyuan, 030001 China; 3 Department of Paediatric Medicine, Shanxi Medical University, Taiyuan, 030001 China

**Correction: Journal of Orthopaedic Surgery and Research (2023) 18:368** **https://doi.org/10.1186/s13018-023-03848-9**

Following publication of the original article [1], the authors identified some errors in the authors’ affiliations. The corrected affiliations are given below.

Jian Li^1+^, Xiuyu Qin^2+^, Jiani Wang^3^, Wangzhe Yang^2^, Junjun Bai^2^, Jia Lv^2^*

^1^Department of Orthopaedics, Third Hospital of Shanxi Medical University, Shanxi Bethune Hospital, Shanxi Academy of Medical Sciences, Tongji Shanxi Hospital, Taiyuan, 030032, China

^2^Department of Orthopaedics, The Second Hospital of Shanxi Medical University, Taiyuan, 030001, China

^3^Department of Paediatric Medicine, Shanxi Medical University, Taiyuan, 030001, China

The original article has been corrected.
